# SPHK1 potentiates colorectal cancer progression and metastasis via regulating autophagy mediated by TRAF6-induced ULK1 ubiquitination

**DOI:** 10.1038/s41417-023-00711-1

**Published:** 2023-12-22

**Authors:** Da Chen, Jiangni Wu, Xinze Qiu, Shibo Luo, Shanpei Huang, Erdan Wei, Mengbin Qin, Jiean Huang, Shiquan Liu

**Affiliations:** 1 Department of Gastroenterology, The Second Affiliated Hospital of Guangxi Medical University, Nanning, Guangxi P. R. China; 2 Department of Pathology, The Second Affiliated Hospital of Guangxi Medical University, Nanning, Guangxi P. R. China

**Keywords:** Colorectal cancer, Cell biology, Colorectal cancer, Metastasis

## Abstract

A sphingolipid metabolite regulator, sphingosine kinase 1 (SPHK1), plays a critical role in the development of colorectal cancer (CRC). Studies have demonstrated that invasion and metastasis of CRC are promoted by SPHK1-driven autophagy. However, the exact mechanism of SPHK1 drives autophagy to promote tumor progression remains unclear. Here, immunohistochemical detection showed the expression of SPHK1 and tumor necrosis factor receptor-associated factor-6 (TRAF6) in human CRC tissues was stronger than in adjacent normal tissues, they were both associated with distance metastasis. It was discovered that knockdown of SPHK1 reduced the expression of TRAF6, inhibited autophagy, and inhibited the growth and metastasis of CRC cells in vitro. Moreover, the effects of SPHK1-downregulating were reversed by overexpression of TRAF6 in CRC cells transfected by double-gene. Overexpression of SPHK1 and TRAF6 promoted the expression of autophagy protein LC3 and Vimentin, while downregulated the expression of autophagy protein P62 and E-cadherin. The expression of autophagy-related ubiquitination protein ULK1 and Ubiquitin protein were significantly upregulated in TRAF6-overexpressed CRC cells. In addition, autophagy inhibitor 3-methyladenine (3MA) significantly inhibited the metastasis-promoting effect of SPHK1 and TRAF6, suppressed the expression of LC3 and Vimentin, and promoted the expression of P62 and E-cadherin, in CRC cells. Immunofluorescence staining showed SPHK1 and TRAF6 were co-localized in HT29 CRC cell membrane and cytoplasm. Immunoprecipitation detection showed SPHK1 was efficiently combined with the endogenous TRAF6, and the interaction was also detected reciprocally. Additionally, proteasome inhibitor MG132 treatment upregulated the expression of TRAF6 and the level of Ubiquitin protein, in SPHK1-downregulating CRC cells. These results reveal that SPHK1 potentiates CRC progression and metastasis via regulating autophagy mediated by TRAF6-induced ULK1 ubiquitination. SPHK1-TRAF6-ULK1 signaling axis is critical to the progression of CRC and provides a new strategy for the therapeutic control of CRC.

## Introduction

Colorectal cancer (CRC) is the most prevalent malignant tumor of digestive system and accounts for ~9% of tumor-related deaths worldwide. The main cause of poor prognosis in CRC is invasion and metastasis accounting for 50% of CRC patients who will eventually experience recurrence, which will shorten the survival of patients and affect their quality of life [1, 2]. Therefore, it’s urgently necessary to investigate invasion and metastasis mechanisms and find out the novel therapeutic targets of CRC.

Sphingosine kinase 1 (SPHK1) is a vital factor that regulates the dynamic balance of ceramide, sphingolipid, and sphingolipid-1-phosphate produced by sphingolipid catabolic metabolism. As a regulatory factor, it regulates proliferation, differentiation, invasion, migration, as well as other pathophysiological activities [3]. Abnormal expression of SPHK1 not only promotes the transformation, proliferation, and angiogenesis of a variety of human tumors, but also contributes to the degree of malignancy and poor prognosis of tumors [4]. Previous studies have found that the expressions of SPHK1 had a positive correlation with CRC metastasis, and patients with SPHK1-positive cancer had a worse prognosis than those with SPHK1-negative cancer [5]. Further, by enhancing the expression and phosphorylation of focal adhesion paxillin, SPHK1-driven autophagy may facilitate CRC metastasis [6]. Autophagy, a dynamic degradation and recycling system, facilitates tumor growth, survival, and metastasis. It enhances the aggressiveness of cancers [7]. It has been reported that SPHK1, as a differentially expressed gene, is mainly enriched in autophagy-related pathways to predict the clinical prognosis of oral squamous cell carcinoma [8]. SPHK1 enhances the peritoneal spread of gastric cancer by regulating autophagy of peritoneal mesenchymal cells [9], and SPHK1 is targeted by microRNA-506-3p to inhibit osteosarcoma cell autophagy and invasion [10]. However, the exact mechanism of SPHK1 drives autophagy to promote tumor progression remains unclear.

Researchers discovered that SPHK1 promotes autophagy protein ULK1 expression [11]. The initiation of autophagy is regulated by ubiquitination of ULK1 and VPS34 and mediated by E3 ubiquitin ligase, which regulates protein activity through non-degrading ubiquitination by Lys63 or promotes protein degradation through degrading ubiquitination by Lys48 [12, 13]. Ubiquitination proteins are a post-translational modification that affects the cellular fate of proteins. The addition of ubiquitin to proteins is performed by the sequential action of three enzymes: E1, ubiquitin-activating enzyme, E2, ubiquitin-conjugating enzyme, and E3, ubiquitin ligase. The tumor necrosis factor receptor-associated factors (TRAF) family protein belongs to a really interesting new gene (RING)-type E3 ubiquitin ligase [14]. Interestingly, TRAF6, as an important E3 ubiquitin ligase, catalyzes Lys63 ubiquitination of ULK1, Beclin1, and other autophagy proteins during the formation of double-membrane autophagosomes, maintaining the stability of both proteins and promoting autophagy [15, 16]. What’s more, AMPKα1 positively regulates the progression of lung cancer and breast cancer by regulating the TRAF6-BECN1 signaling axis [17]. Thus, TRAF6 appears to be a key regulator of autophagy-promoting tumor metastasis.

However, it is unclear whether SPHK1 relies on TRAF6 to induce autophagy and thus promote tumor metastasis in CRC. This study focused on elucidating SPHK1 regulates autophagy, and subsequently focused on the TRAF6-induced ULK1 ubiquitination relying on the combination of SPHK1 and TRAF6. In the present study, findings highlight that autophagy mediated by TRAF6-induced ULK1 ubiquitination played a critical role in CRC metastasis potentiated by SPHK1, providing the SPHK1-TRAF6-ULK1 signaling axis is an underlying novel therapeutic target for CRC.

## Materials and methods

### Patients tissue samples

Ninety tumor tissues and corresponding normal tissues were explored in this study. The age range of the patients with CRC was 32 to 93. The National Comprehensive Cancer Network classified the patients as follows: 7 patients with I stage, 43 patients with II stage, 28 patients with III stage, and 12 patients with IV stage.

Inclusion criteria: (1) no preoperative chemotherapy or radiotherapy; (2) patients underwent radical resection of CRC and were diagnosed with CRC by postoperative pathological examination. Exclusion criteria: (1) combined with other tumors; (2) patients with chronic diseases; (3) patients with drug history. All the above tissue specimens were collected with the informed consent of the patients, and this study was approved by the Medical Ethics Committee of the Second Affiliated Hospital of Guangxi Medical University (No. 2021-0274).

### Cell culture and transfection

CRC cell lines (RKO, HT29) were purchased from the Cell Bank of Shanghai Institute of Biological Science (SIBS, CAS, China) with STR (short tandem repeat) appraisal certificates. And the cell lines were tested for mycoplasma contamination. SPHK1- shRNA or TRAF6- shRNA lentivirus was transfected into RKO cells. HT29 cells were infected with SPHK1 overexpression, TRAF6 overexpression, and blank lentiviral vectors. All stable subsets of transfected cells were nurtured in DMEM media (Gibco, USA) containing 10% fetal bovine serum (FBS, Gibco, USA) and 1% penicillin–streptomycin mixture (Gibco, USA) at 37 °C, in 5% CO_2_.

### Reagents and antibodies

CCK8 kit (C6005) was purchased from New Cell&Molecular Biotech Co.,Ltd (Suzhou, China). Anti-LC3 I/II (M186-3) was purchased from MBL (Boston, MA, USA), anti-SPHK1 (ab71700) was purchased from Abcam (Cambridge, UK), anti-TRAF6 (8028), E-cadherin (3195), ULK1(D8H5) and Vimentin (5741) were purchased from Cell Signaling Technology (Beverly, MA, USA), anti-SPHK1 (10670-1-AP) and anti-TRAF6 (CL488-66498) was purchased from Proteintech (Wuhan, China), anti-Ubiquitin (13-1600) was purchased from Thermo Fisher Scientific (MA, USA) and GAPDH (21612) was purchased from SAB (California, USA). 3-Methyladenine (3MA, HY-19312) was purchased from MedChemExpress (China).

### Immunohistochemistry detection

CRC tissues were fixed in formalin, embedded in paraffin, and sectioned. Sections were further used for immunohistochemical staining. According to IHC, positive staining was quantified and classified into 4 categories: 1, 26–50% scored as 2, and >50% scored as 3. Staining intensity was graded as: 0, achromatic, 1, light yellow, 2, yellow, and 3, brown. All scores were independently reviewed by two pathologists. The two scores were added for the final score: 0 score: (−), 1–2 score: (+), 3–4 score: (++), and a score of 5 or above: (+++). The median of the IHC score was chosen as the cut-off value for the high (>3) and low (≤3).

### Immunofluorescence

The cell slides were processed and stained with antibodies to detect SPHK1 and TRAF6. The slides were nurtured with the corresponding secondary antibodies and the nuclei were stained with DAPI.

### Co-immunoprecipitation

The cells were washed in cold PBS before being used in the lysis buffer. Cell lysates were prepared by incubation with protein A/G beads for 1 h at 4 °C. IP beads were prepared by incubating 30 µl protein A/G beads with 5 µG antibodies for 2 h at 4 °C. IPs were performed overnight at 4 °C and bound proteins were eluted by boiling in SDS protein loading buffer for 5 min. IP cleavage products were subjected to Western blotting with the indicated antibodies. Anti-SPHK1 (10670-1-AP) was purchased from Proteintech (Wuhan, China), anti-TRAF6 (8028) were purchased from Cell Signaling Technology (Beverly, MA, USA). CO-IP kit (P2179) was purchased from Beyotime (Shanghai, China).

### Western blot

Cells were homogenized and lysed, and protease inhibitors (Solarbio, China) were added for protein quantification using the BCA method (Beyotime, China). Cellular proteins were subjected to electrophoresis under suitable conditions, electroporated, transferred to polyvinylidene fluoride (PVDF, Millipore, USA) membranes, and blocked. The primary antibodies were nurtured overnight with the membranes at 4 °C: SPHK1 (1:1000, Abcam, Proteintech), LC3 (1:1000, MBL), TRAF6 (1:1000, CST), ULK1 (1:1000, CST), P62 (1:1000, CST), E-cadherin (1:1000, CST), Vimentin (1:1000, CST), Ubiquitin (1:1000, TFS) and the secondary antibodies were nurtured for 1 h at room temperature in a shielded light environment. GAPDH (1:10000, SAB) was used as the loading control. With Odyssey CLx Infrared Imaging System (LI-COR Biosciences, Lincoln, NE, USA), images and band intensities were quantified.

### Cell proliferation assay

In 96-well plates, cells were arranged overnight at a density of 5 × 10^3^ per well. After attachment, the viability was determined by adding 10 μl of 5 mg/ml CCK8 solution to every well and incubating for 1–4 h.

At 37 °C with 5% CO_2_, log-phased cells were plated into 6 well plates and fertilized every 3 days with a growth medium. After 14 days of incubation, colonies were fixed with 4% paraformaldehyde, stained with crystal violet, and counted with Image J software.

### Wound healing and transwell assays

Wound healing assays were conducted by scratching cells with a 90% confluence tip, washing them with PBS, and then culturing them for 48 hours in a serum-free medium. For transwell migration assay, 2.5×10^5^ /ml cells were incubated in serum-free medium on top of the transwell filter (8 μm pore size, Corning) while 700 μL 10% FBS was used on the lower chamber of the filter. For transwell invasion assay, 60-80 µl of diluted Matrigel (3.9 µg/µl) was added to the polycarbonate membrane in the upper chamber, and the Matrigel was allowed to polymerize into a gel at 37 °C for 30 min. The cells were passed through the transwell filter containing Matrigel. After 48 h co-culture, following 4% paraformaldehyde fixation, Giesa staining was performed on the cells that were migrated to lower surfaces of the membranes.

### Transmission electron microscope

NC-HT29, SPHK1(+)-HT29, and TRAF6(+)-HT29 cells were fixed, embedded in 1% agarose, and then fixed. The cells were dehydrated with ethanol, infiltrated with proportional acetone and EMbed 812 before being roasted and embedded, and then cut into ultrathin sections. Ultrathin sections were examined by transmission electron microscopy after final staining with uranyl acetate and lead citrate.

### Animal experiments

The animal studies were performed according to the guidelines of the Institutional Animal Care and Use Committee of Guangxi Medical University under approved protocols. For tumor growth assays, 5 × 10^6^ HT29 cells were injected into the dorsal flank of 4-week-old male BALB/c nude mice. Every 2 days, the body weight and tumor volume were monitored. Tumor volume was calculated by the formula *V* = 0.5 × a × b^2^ (a: tumor length, b: tumor width). Eighteen days after inoculation, all mice (*n* = 5, per group) were euthanized for analyses. A formalin fixation and paraffin embedding of tumor tissues were followed by sectioning of the paraffin samples. Tumor sections were used for immunohistochemistry to further detect SPHK1, TRAF6, LC3, P62, ULK1, and Ubiquitin expression. The Tab of Animal Experimental Ethical Inspection: NO. 202209001.

### Statistical analysis

Experiments were repeated at least three times independently, and measurements were expressed as mean ± SD. The chi-square test was used to compare binary variables, and Fisher’s exact test was used for comparison between groups. SPSS 22.0 software was used for t-test or analysis of variance. *P* < 0.05 was considered statistically significant.

## Results

### Expression and clinicopathological features of SPHK1 and TRAF6 in CRC tissues

**Fig. 1:**
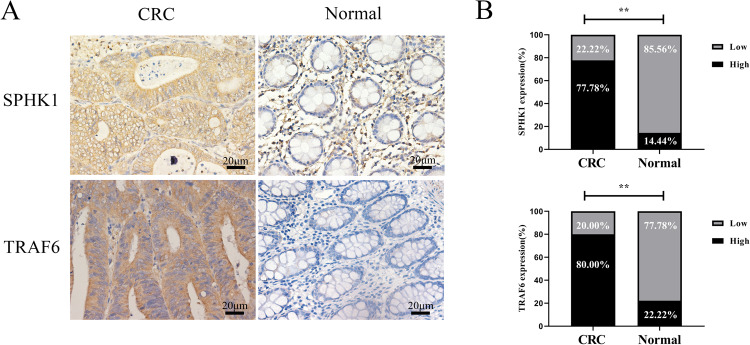
Expression of SPHK1 and TRAF6 in CRC and adjacent normal colon tissues. **A** Representative images of different cytoplasm SPHK1 and TRAF6 expressions in CRC and adjacent normal colon tissues. Scale bar, 20 µm. **B** Cytoplasm SPHK1 and TRAF6 expression was upregulated in CRC tissues compared to adjacent normal colon tissues (*n* = 90, paired *t* test). **P < 0.01, based on the Chi-squared test.

SPHK1 and TRAF6 expressions in CRC tissues were significantly higher than those in adjacent normal tissues (Fig. 1). Metastasis development was significantly correlated with SPHK1 and TRAF6 expression (Table 1). Additionally, SPHK1 expression in CRC tissues was closely related to TRAF6 expression (Table 2). Collectively, these results indicated that SPHK1 and TRAF6 were key factors that promote CRC formation and metastasis.

**Table 1:** The expression and clinicopathological features of SPHK1 and TRAF6 in human CRC tissues.

		SPHK1 expression			TRAF6 expression		
Pathologic feature	*N*	Low	High	*P*	Low	High	*P*
Infiltration depth							
Mucosa and superficial muscular layer	18	5	13	0.536	4	14	0.751
Deep muscular layer and below	72	15	57		14	58	
Sex							
Male	50	7	43	0.036	9	41	0.596
Female	40	13	27		9	31	
TNM staging							
I+II stage	50	8	42	0.112	7	43	0.112
III+IV stage	40	12	28		11	29	
Lymphatic metastasis							
−	63	12	51	0.268	13	50	0.818
+	27	8	19		5	22	
Distant metastasis							
−	77	14	63	0.035	12	65	0.020
+	13	6	7		6	7

**Table 2:** Correlation between the expression of SPHK1 and TRAF6 in colorectal cancer tissues.

Tumor	SPHK1 low	SPHK1 high	Correlation	*P* value
TRAF6 low	13	5	0.601	0.000
TRAF6 high	7	65		

### SPHK1 promoted TRAF6 expression in CRC cells

**Fig. 2:**
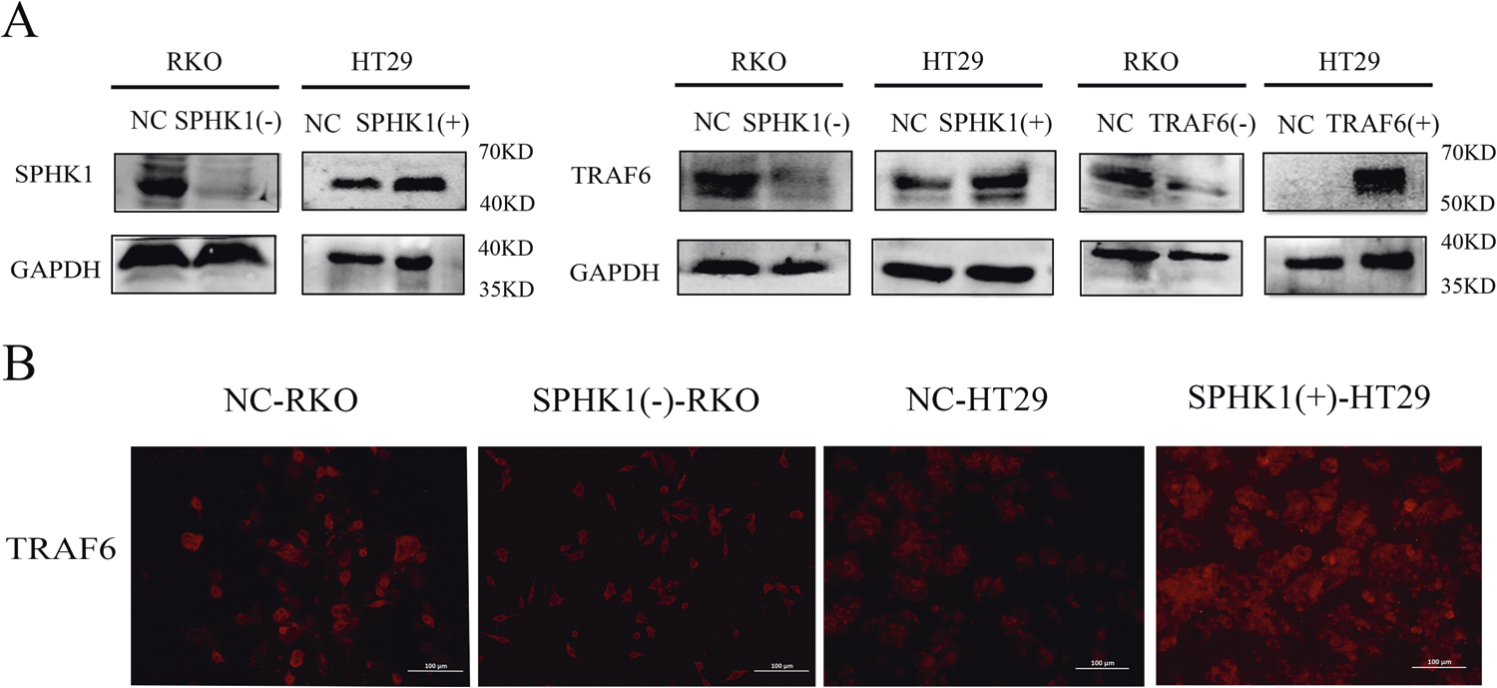
The expression of SPHK1 and TRAF6 in CRC cells. **A** The efficiency of SPHK1 overexpression and knockdown was measured by western blot (left). The changes in the expression of TRAF6 level following SPHK1 overexpressed or down-regulated in HT29 or RKO cells identified by western blot (right). **B** Immunofluorescence assay revealed that SPHK1 regulated the level of TRAF6. Scale bar, 100 µm. Data were presented by mean with SD (*n* ≥ 3). *P < 0.05, **P < 0.01.

Western blot confirmed that RKO cells were infected with SPHK1 shRNA or TRAF6 shRNA lentivirus particles, and HT29 cells were stably infected with SPHK1 and TRAF6 overexpression vectors. As showed in Fig. 2A, SPHK1(-)-RKO, TRAF6(-)-RKO, SPHK1(+)-HT29 and TRAF6(+)-HT29 cell lines were constructed. Moreover, the expression levels of TRAF6 protein were significantly decreased when SPHK1 was knocked down, while overexpression of SPHK1 resulted in a significant increase in the protein expression level of TRAF6 (Fig. 2A, B).

### SPHK1 protein regulated TRAF6 protein expression in CRC cells

**Fig. 3:**
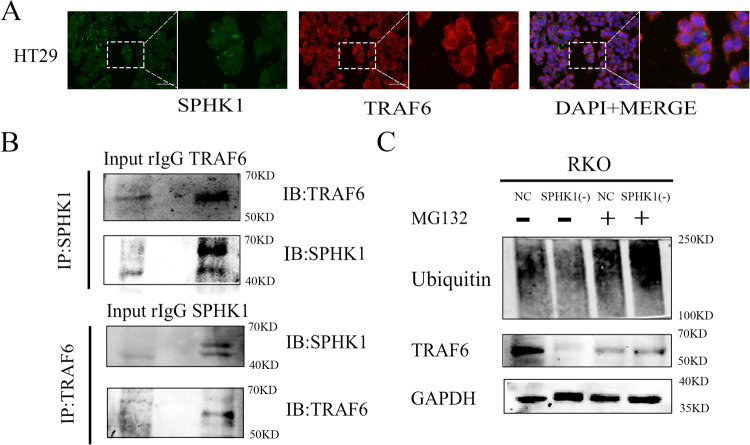
SPHK1 is a TRAF6-interacting protein in CRC cells. **A** Immunofluorescence staining showed that SPHK1 and TRAF6 were expressed in the cytoplasm of HT29 cells. Scale bar, 50 µm. **B** Western blot analyses of the co-immunoprecipitations between endogenous SPHK1 and TRAF6. Cell extracts from HT29 cell line were immunoprecipitated with nonspecific IgGs and indicated antibodies. **C** NC-RKO and SPHK1(-)-RKO cells were treated with or without 10 µM MG132 for 6 h, followed by Western blot with antibodies as indicated. Ub ubiquitin. Data were presented by mean with SD (*n* ≥ 3).

To determine the subcellular distribution of SPHK1 and TRAF6 in HT29 cells, Immunofluorescence staining revealed that SPHK1 and TRAF6 were co-localized in the cell membrane and cytoplasm (Fig. 3A). Co-IP assays were performed to check the interaction between SPHK1 and TRAF6 in HT29 cells. It’s shown that SPHK1 could co-immunoprecipitate with TRAF6, and TRAF6 also could co-immunoprecipitate with SPHK1 (Fig. 3B). As TRAF6 protein degradation is mediated by the proteasome pathway, to explore the mechanism of down-regulated SPHK1 induced TRAF6 reduction, SPHK1(-)-RKO cells were treated with proteasome inhibitor (MG132). TRAF6 expression was upregulated, accompanied by increased Ubiquitin expression (Fig. 3C). These results suggest that SPHK1 may inhibit the degradation of TRAF6 protein via stabilizing protein activity by ubiquitination.

### SPHK1 and TRAF6 enhanced CRC cells growth and metastasis

**Fig. 4:**
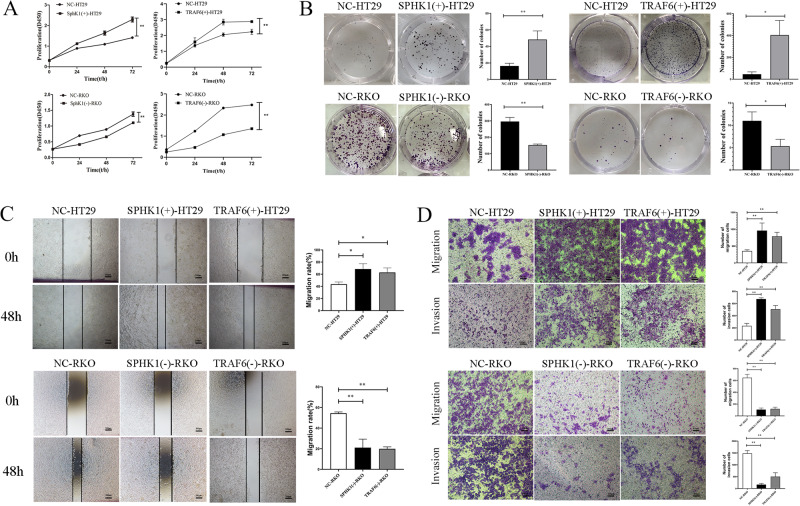
SPHK1 and TRAF6 enhanced the proliferation and metastasis of CRC cells. SPHK1 and TRAF6 both promoted CRC cell growth, evaluated by CCK8 (**A**) and colony formation assays (**B**). SPHK1 and TRAF6 both promoted CRC cell migration and invasion, evaluated by wound healing assay (**C**, Scale bar, 200 µm) and transwell (**D**, Scale bar, 200 µm). Data were presented by mean with SD (*n* ≥ 3). *P < 0.05, **P < 0.01.

As compared to NC-HT29 group, SPHK1(+)-HT29 and TRAF6(+)-HT29 cells showed significantly increased cell viability and cloning abilities, while SPHK1(-)-RKO and TRAF6(-)-RKO cells showed significantly decreased viability. (Fig. 4A, B), indicating that both SPHK1 and TRAF6 enhanced CRC cell growth in vitro.

As compared to NC-HT29 group, the wound healing rate and the number of migrated and invaded cells increased in SPHK1(+)-HT29 and TRAF6(+)-HT29 groups, while the wound healing rate and the number of migrated and invaded cells decreased in SPHK1(-)-RKO and TRAF6(-)-RKO groups (Fig. 4C, D). These results indicated that both SPHK1 and TRAF6 could promote the metastasis ability of CRC cells.

### SPHK1 and TRAF6 induced the autophagy flux of CRC cells

**Fig. 5:**
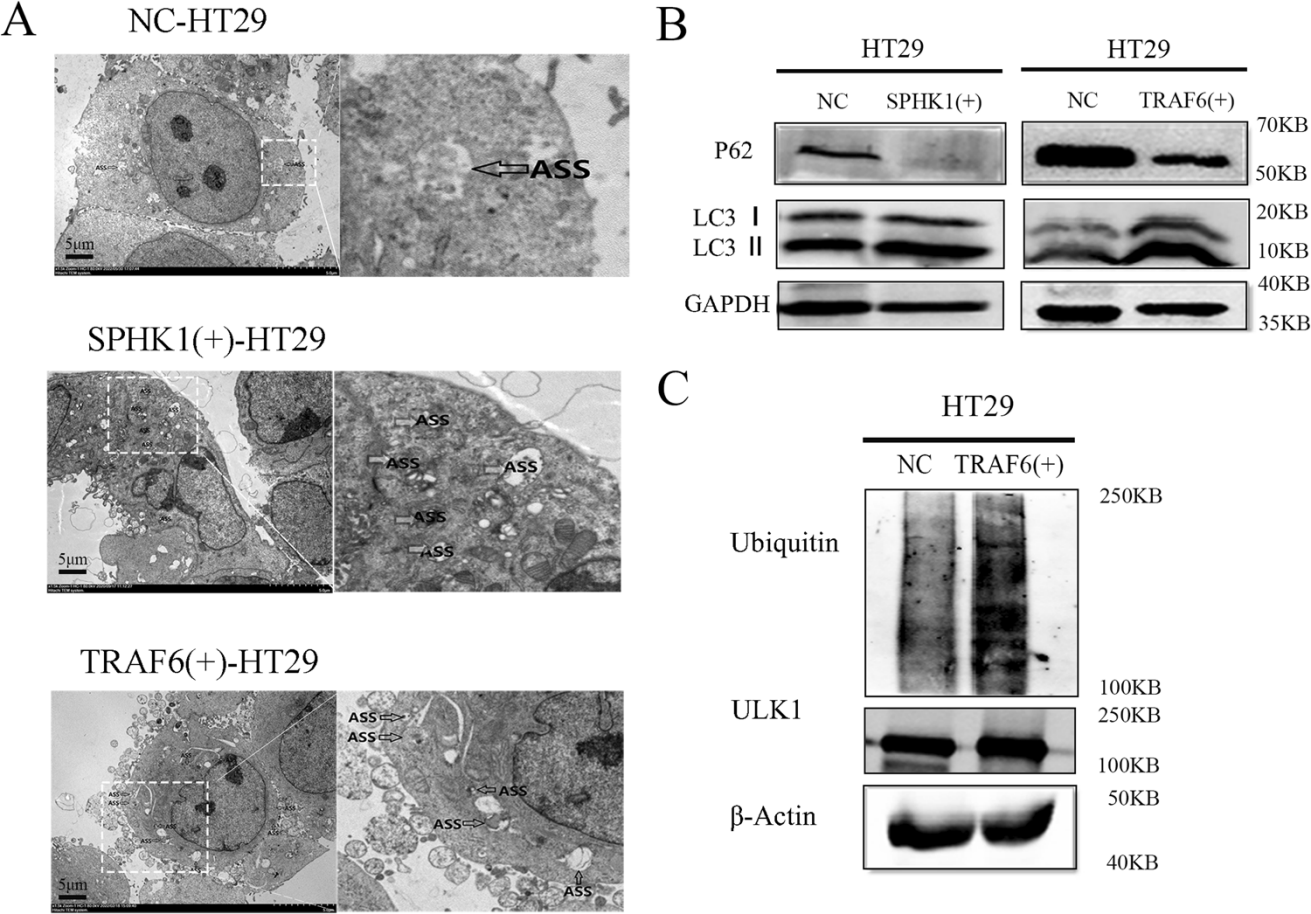
SPHK1 and TRAF6 induced the autophagy flux of CRC cells. **A** SPHK1 and TRAF6 are involved in induced autophagy in CRC cells. Transmission electron microscopy showing the formation of autophagy flux in NC-HT29, SPHK1(+)-HT29, and TRAF6(+)-HT29 cells. The arrow shows the autophagosomes/autolysosomes. Scale bar, 5 µm. **B** SPHK1 and TRAF6 promoted the expression of autophagy protein LC3, inhibited p62 protein, evaluated by western blot. **C** TRAF6 promote the expression of autophagy-related ubiquitination protein ULK1 and its ubiquitin level in TRAF6(+)-HT29 cells, evaluated by western blot. Data were presented by mean with SD (*n* ≥ 3). *P < 0.05, **P < 0.01.

As compared to NC-HT29 group, SPHK1(+)-HT29 and TRAF6(+)-HT29 groups significantly induced the autophagy flux in CRC cells (Fig. 5A). The expression of autophagy protein LC3 II/I was enhanced and the expression of autophagy protein P62 was down-regulated in SPHK1(+)-HT29 and TRAF6(+)-HT29 groups (Fig. 5B). In TRAF6(+)-HT29 cells, the expression of autophagy-related ubiquitination protein ULK1 and Ubiquitin protein were significantly upregulated (Fig. 5C).

### Autophagy was required in promoting cell metastasis and EMT in CRC cells

**Fig. 6:**
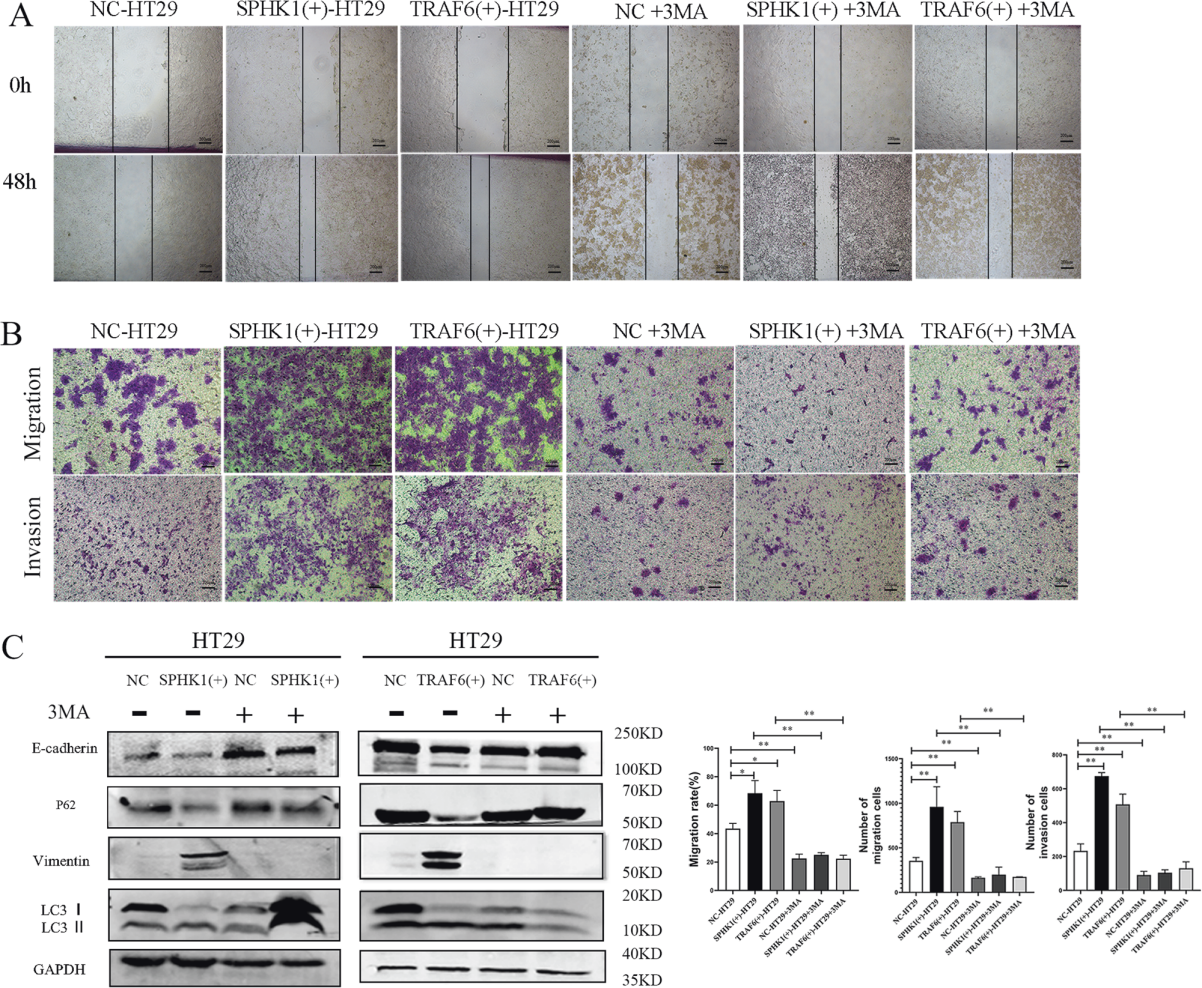
Effect of autophagy inhibitor 3MA on the metastasis ability in SPHK1(+)-HT29 and TRAF6 (+)-HT29 groups cells. Metastasis of CRC cells was inhibited by 3MA in SPHK1(+)-HT29 and TRAF6(+)-HT29 cells, evaluated by wound healing assay (**A**, Scale bar, 200 µm) and transwell (**B**, Scale bar, 200 µm). **C**. Autophagy protein and EMT-related protein were inhibited by 3MA in SPHK1(+)-HT29 and TRAF6(+)-HT29 cells, evaluated by Western blot. (3MA: 3-Methyladenine, 5 mM, 48 h). Data were presented by mean with SD (*n* ≥ 3). *P < 0.05, **P < 0.01.

Since SPHK1 and TRAF6 promote autophagy and metastasis of CRC cells, respectively, the relationship between autophagy and metastasis was investigated further. NC-HT29, SPHK1(+)-HT29 and TRAF6(+)-HT29 cells were treated with autophagy inhibitor 3MA. As shown in Fig. 6A, B, compared with untreated cells, there was a significant difference in wound healing rate and the number of migrated and invaded cells in SPHK1(+)-HT29 and TRAF6(+)-HT29 cells after being treated with 3MA. Meanwhile, compared with NC-HT29 cells after being treated with 3MA, there was no significant change in wound healing rate and the number of migrated and invaded cells in SPHK1(+)-HT29 and TRAF6(+)-HT29 cells after being treated with 3MA. Results showed 3MA treatment attenuated the original ability of SPHK1 and TRAF6 to promote cell metastasis.

Compared with NC group cells, 3MA significantly inhibited the expression of autophagy protein LC3 II/I and upregulated autophagy protein P62 in SPHK1(+)-HT29 and TRAF6(+)-HT29 cells, indicated the autophagy level was signally reduced. Furthermore, compared with NC-HT29 cells after being treated with 3MA, 3MA significantly enhanced the expression of E-cadherin and down-regulated the expression of Vimentin in SPHK1(+)-HT29 and TRAF6 (+)-HT29 cells (Fig. 6C). In summary, SPHK1 and TRAF6 may promote EMT and metastasis of CRC cells through autophagy.

### TRAF6 overexpression reversed the effects of SPHK1 knockdown on tumor cells proliferation, metastasis, and autophagy

**Fig. 7:**
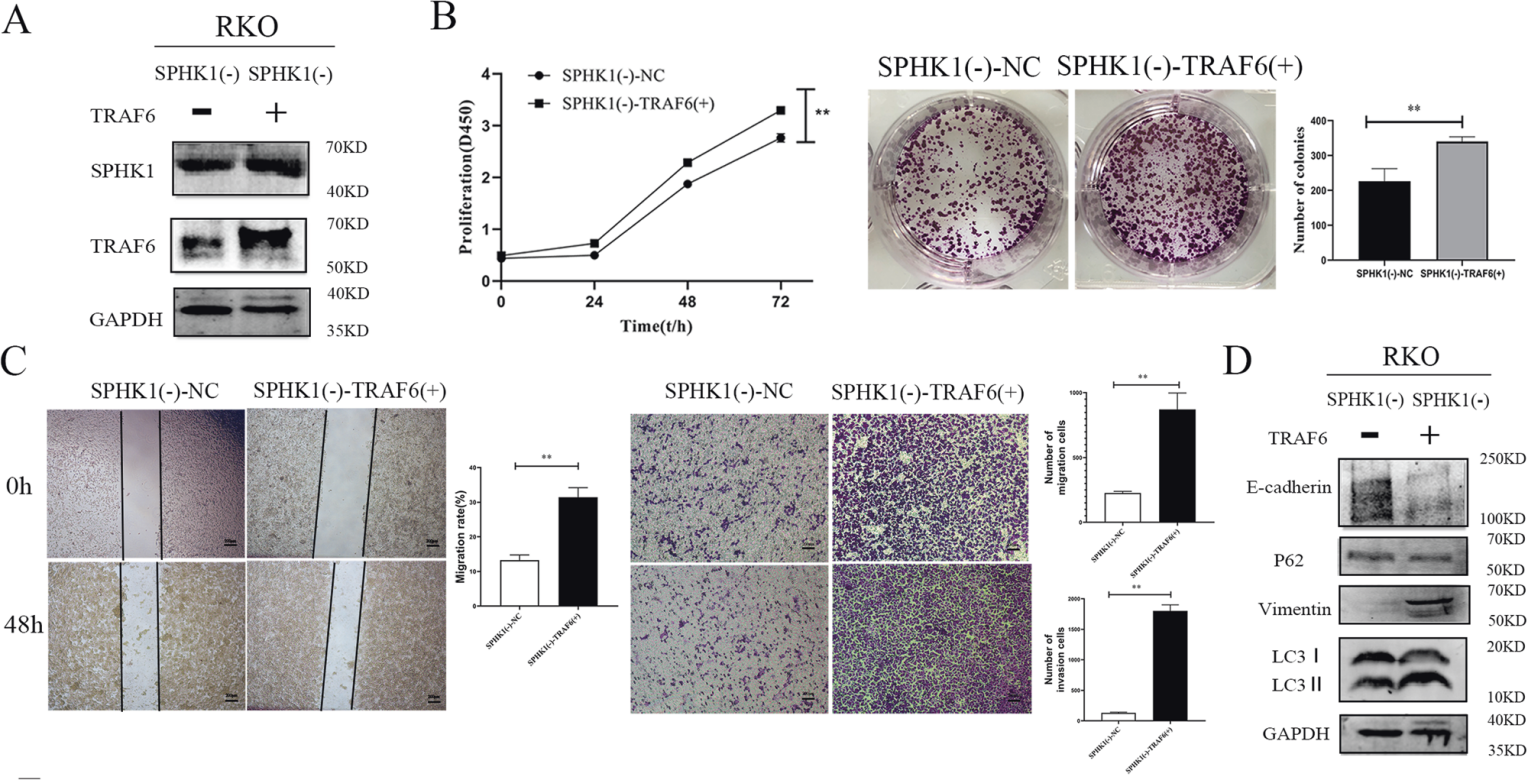
The inhibitory effect of SPHK1 knockdown on CRC cells proliferation and metastasis was rescued by the overexpression of TRAF6. **A** Dual-gene transfection cells of SPHK1(-)-NC-RKO and SPHK1(-)-TRAF6(+)-RKO were successfully constructed, and evaluated by western blot. **B** Overexpression of TRAF6 enhanced SPHK1 knockdown induced suppression of CRC cell growth, evaluated by CCK8 and colony formation assays. **C** Overexpression of TRAF6 enhanced SPHK1 knockdown induced suppression of CRC cell metastasis, evaluated by wound healing assay and transwell. Scale bar, 200 µm. **D** Overexpression of TRAF6 rescued the expression of autophagy and EMT proteins in SPHK1(-)-RKO cells, evaluated by Western blot. Data were presented by mean with SD (*n* ≥ 3). *P < 0.05, **P < 0.01.

TRAF6 overexpression vector was stably transfected into SPHK1(-)-RKO cells. And it showed the SPHK1(-)-TRAF6(+) cell model was successfully constructed (Fig. 7A). CCK8 and plate cloning detection showed the growth and migratory ability of double-gene transfection SPHK1(-)-TRAF6(+)-RKO cells were significantly stronger than those of SPHK1(-)-NC-RKO group cells (Fig. 7B, C). Compared with control group, the expression of LC3 II/I, ULK1, and Vimentin increased, while the expression of E-cadherin and P62 was down-regulated in SPHK1(-)-TRAF6(+)-RKO group cells (Fig. 7D).

### SPHK1 and TRAF6 were required for tumor growth in vivo

**Fig. 8:**
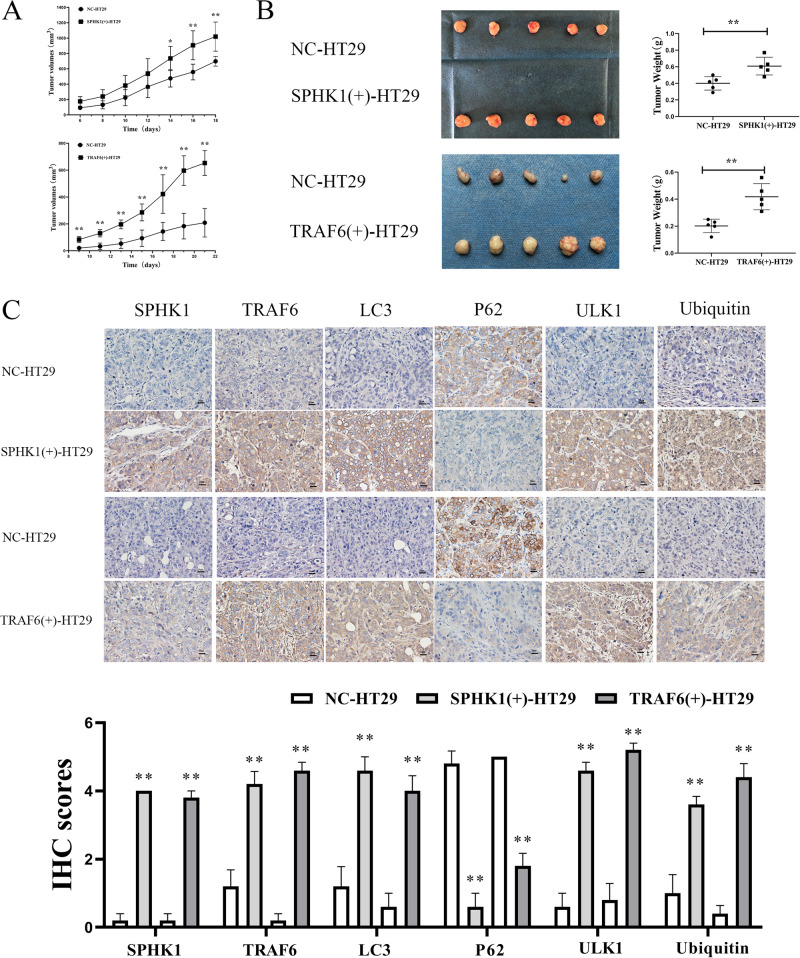
SPHK1 and TRAF6 promoted the proliferation of CRC cells in vivo. SPHK1 and TRAF6 overexpression promoted the proliferation of CRC cells in nude mice. Tumors volume (**A**) and tumor weight (**B**) were measured after subcutaneous injection of NC-HT29, TRAF6(+)-HT29, and SPHK1(+)-HT29 cells. **C** High expression of SPHK1, TRAF6, LC3, ULK1, and Ubiquitin in SPHK1(+)-HT29 and TRAF6(+)-HT29 group, while P62 expression was low. Immunohistochemical staining of TRAF6, LC3, P62, ULK1, and Ubiquitin in subcutaneous tumor tissues. (vs. NC-HT29 group, 5 mice per group. Scale bar, 20 µm. Data are shown as the mean ± SD. *n* ≥ 3). *P < 0.05, **P < 0.01.

NC-HT29, SPHK1(+)-HT29, and TRAF6(+)-HT29 cells were used to establish mice models. Mice were injected subcutaneously with the designated cells, and tumors in the SPHK1(+)-HT29 and TRAF6(+)-HT29 groups developed significantly faster than that of NC-HT29 group (Fig. 8A). Further, the tumors of the SPHK1(+)-HT29 and TRAF6(+)-HT29 group were significantly heavier compared with NC-HT29 group (Fig. 8B). Moreover, with the up-regulation of SPHK1 and TRAF6, the expressions of LC3 II/I, ULK1, and Ubiquitin were increased in tissues of subcutaneous tumors, while P62 was decreased (Fig. 8C). These results indicated that SPHK1 and TRAF6 promoted tumor growth in vivo.

## Discussion

Colorectal cancer (CRC) is one of the most common malignant tumors in humans [1]. Invasion and metastasis are the main cause of CRC-related death, and there is no effective treatment at present. Studies have shown that autophagy plays a promoting role in the formation and development of tumors. Autophagy is a process that degrades damaged proteins or organelles in the cell in order to maintain the intracellular environment’s homeostasis [18]. Autophagy was involved in the process of precancerous cell lesions, epithelial-mesenchymal transition (EMT), cell invasion and metastasis, tumor immune microenvironment, etc [19]. Inhibition of autophagy can regulate the interaction between LC3 and paxillin, promote the formation of cell-cell focal adhesion (FA), and thus inhibit the invasion and metastasis of breast cancer, lung cancer, and liver cancer cells [20]. Studies have also shown that autophagy is activated in CRC, and autophagy may promote the survival of cancer cells in the tumor microenvironment [21]. Our previous study found that SPHK1 induced EMT through FAK/AKT/MMPs and promoted migration and metastasis of CRC [5]. We also found that by enhancing the expression and phosphorylation of the FA protein paxillin, SPHK1-driven autophagy may facilitate CRC metastasis [6]. During FA turnover, paxillin participates as a scaffolding protein in the rapid assembly and disassembly of FA [22]. At the front of migrating cells, the dynamic conversion of FA is the key to effective cell migration [23, 24]. When FA was reduced with the knockdown of integrin-linked kinase (ILK), downregulation of FA resulted in a shift from cell-matrix adhesion to cell-cell adhesion, and low cell-matrix adhesion decreased EMT [25]. In the present study, the cell migration-promoting ability and the expression of EMT-related protein mediated by SPHK1 overexpression were reversed by autophagy inhibitor 3MA in CRC cells. Therefore, SPHK1 may facilitate the invasion and metastasis of CRC by regulating autophagy-mediated EMT. However, the mechanism of SPHK1 regulates autophagy and then mediates EMT in CRC invasion and metastasis has not been elucidated.

An autophagy initiation process begins with the activation of ULK1 and its ubiquitination, mediated by E3 ubiquitin ligase [26]. E3 ubiquitin ligase was used to stabilizing protein activity through non-degradative ubiquitination or promoting protein degradation through degradative ubiquitination [12, 13]. E3 ligases fall into three classes: really interesting new gene (RING) E3s, homology to E6AP C terminus (HECT) E3s, and RING-between-RING (RBR) E3s. Each of these classes has conserved structural domains and a mechanism by which ubiquitin is transferred from the E2 to the substrate. Ubiquitin is directly transferred from the E2 enzyme to the substrate by the RING family, which simultaneously binds both E2~Ub thioester and substrate [27, 28]. Tumor necrosis factor receptor-associated factors6 (TRAF6), a specific RING finger E3 ubiquitin ligase, has been proposed to mediate the Lys63 ubiquitination of ULK1 and Beclin1 contributing to autophagy-related proteins activation, which induces autophagy [15, 16, 29]. In present study, TRAF6 promoted the occurrence of autophagy by promoting the expression of ubiquitinated protein ULK1 and Ubiquitin protein. Moreover, it acted as a tumor-promoting factor in CRC, promoting tumor proliferation and metastasis. Studies have confirmed that TRAF6 not only induces autophagy to regulate EMT of gastric cancer cells, but also affects CRC migration, invasion, and lymphatic metastasis through its ubiquitination site 124mut [30, 31]. TRAF6-mediated AKT ubiquitination activated AKT to promote CRC growth [32]. Besides, it promoted the EMT of HCC by enhancing AKT phosphorylation and increasing AKT activity against GSK-3β [33]. In the present study, it was verified that autophagy inhibitor 3MA attenuated the migration ability of TRAF6-overexpressing CRC cells. Therefore, TRAF6 may play a vital role in CRC metastasis by regulating ubiquitination-autophagy-mediated EMT.

Previous studies have found that SPHK1 and TRAF2 interact in HCC. The binding of SPHK1 catalytic product S1P to TRAF2 can promote TRAF2-catalyzed ubiquitination of Beclin1 Lys63 chain and promote autophagy and EMT [34]. However, our co-immunoprecipitation assay showed that SPHK1 and TRAF6 interact with each other in CRC. TRAF6 is a ubiquitin ligase similar to TRAF2, depending on the integrity of their RING finger domains [35]. Structural differences are multifaceted, including amino acid differences at key Ubc13 interaction sites, as well as local conformational differences [36]. The TRAF6 and TRAF2 proteins function as ubiquitin E3 ligases that synthesize Lys63-linked polyubiquitin chains and activate protein kinases independently of the proteasome [36]. In CRC tissues, SPHK1 expression was closely related to TRAF6 expression. The co-localization and interaction of SPHK1 and TRAF6 expression were confirmed by immunofluorescence and immunoprecipitation. Interestingly, SPHK1 knockdown has an inhibitory effect on TRAF6 expression, rescue experiments showed that overexpression of TRAF6 reversed the silencing effect of SPHK1 in SPHK1(-)-RKO cells, indicating that TRAF6 is the downstream factor of SPHK1. So, SPHK1 and TRAF6 as tumor promote factors are closely related in CRC, and SPHK1 may regulate the expression of TRAF6.

In this study, treatment of SPHK1 knockdown cells with the proteasome inhibitor MG132 resulted in the recovery of TRAF6 expression and increased ubiquitination level, indicating that SPHK1 might act to inhibit the degradation of TRAF6 protein (ubiquitination stabilized protein activity). In addition, our previous study found that the FAK/AKT/MMP axis is involved in SPHK1-mediated migration and metastasis of CRC [5]. Studies showed that TRAF6-mediated AKT ubiquitination activates AKT to promote CRC growth, and TRAF6 promotes the EMT of HCC by enhancing AKT phosphorylation [32, 33]. So, AKT may act as a key factor in SPHK1 regulating the stabilized activity protein expression of TRAF6 in CRC.

Our previous study confirmed that SPHK1 could modulate the expression of autophagy protein ULK1/LC3, thereby inducing autophagy in CRC cells [11]. Studies have shown that TRAF6 induces ubiquitination of ULK1 by Lys63 chain, which stabilizes the expression of autophagy protein ULK1 and promotes the occurrence of autophagy, thereby promoting the progression of malignant tumors [15]. In this study, it was found that up-regulation of SPHK1 could enhance TRAF6 expression. Moreover, up-regulation of TRAF6 could also promote ubiquitination protein ULK1 and Ubiquitin protein to active autophagy in vitro and vivo. Therefore, SPHK1 potentiates CRC progression and metastasis via regulating autophagy mediated by TRAF6-induced ULK1 ubiquitination. Further study indicated the cell migration-promoting ability and the expression of EMT-related protein mediated by SPHK1 and TRAF6 overexpression was reversed by autophagy inhibitor 3MA in CRC cells. In CRC, activated autophagy may promote the survival of cancer cells in the tumor microenvironment [21], while inhibiting EMT and invasion of CRC cells by inhibiting hypoxia-induced autophagy [37].

In conclusion, SPHK1 potentiates CRC progression and metastasis via regulating autophagy mediated by TRAF6-induced ULK1 ubiquitination. However, the specific mechanism of SPHK1-TRAF6-ULK1 dependent autophagy promotes CRC metastasis still needs to be further studied.

## Data Availability

The datasets used and/or analyzed during the current study are available upon reasonable request.
